# Socio-demographic and environmental determinants of under-5 stunting in Rwanda: Evidence from a multisectoral study

**DOI:** 10.3389/fpubh.2023.1107300

**Published:** 2023-03-14

**Authors:** Chester Kalinda, Million Phri, Maria Albin Qambayot, Marie Consolatrice Sage Ishimwe, Alemayehu Gebremariam, Abebe Bekele, Rex Wong

**Affiliations:** ^1^Bill and Joyce Cummings Institute of Global Health, University of Global Health Equity, Kigali, Rwanda; ^2^School of Humanities and Social Sciences, University of Zambia, Great East Road Campus, Lusaka, Zambia; ^3^Centre for One Health, University of Global Health Equity, Kigali, Rwanda; ^4^Institute of Global Health Equity Research, University of Global Health Equity, Kigali, Rwanda; ^5^Catholic Relief Services, Rwanda Country Program, Kigali, Rwanda; ^6^School of Medicine, University of Global Health Equity, Kigali, Rwanda

**Keywords:** childhood stunting, multilevel analysis, undernutrition, under-five children, Rwanda

## Abstract

Child stunting is an important household, socio-economic, environmental and nutritional stress indicator. Nationally, 33% of children under 5 in Rwanda are stunted necessitating the need to identify factors perpetuating stunting for targeted interventions. Our study assessed the individual and community-level determinants of under-5 stunting essential for designing appropriate policy and program responses for addressing stunting in Rwanda. A cross-sectional study was conducted between September 6 and October 9, 2022, in five districts of Rwanda including, Kicukiro, Ngoma, Burera, Nyabihu and Nyanza. 2788 children and their caregivers were enrolled in the study and data on the individual level (child, caregiver/household characteristics), and community-level variables were collected. A multilevel logistic regression model was used to determine the influence of individual and community-level factors on stunting. The prevalence of stunting was 31.4% (95% CI: 29.5–33.1). Of this, 12.2% were severely stunted while 19.2% were moderately stunted. In addition, male gender, age above 11 months, child disability, more than six people in the household, having two children below the age of five, a child having diarrhea 1–2 weeks before the study, eating from own plate when feeding, toilet sharing, and open defecation increased the odds of childhood stunting. The full model accounted for 20% of the total variation in the odds of stunting. Socio-demographic and environmental factors are significant determinants of childhood stunting in Rwanda. Interventions to address under-five stunting should be tailored toward addressing individual factors at household levels to improve the nutritional status and early development of children.

## Introduction

Under-five malnutrition is an important sociodemographic, environmental, and healthcare utilization indicator which plays a critical role in influencing the development of healthcare programs and policies (1). A recent joint UNICEF/WHO/World Bank report showed that the global burden of under-five malnutrition remains high with about 149.2 million (22%) being stunted, 45.4 million (6.7%) wasted while 38.9 million (5.7%) are overweight (2). Significant efforts to re-delineate the global nutrition model and make nutrition pivotal in the development agenda have been made. However, regional and country-level disparities remain; with Asia and Africa carrying the heaviest burden, exacerbating the risks of failure to attain the universal right to healthy food as advocated for by the United Nations (3), World Health Assembly target of reducing stunting by 40% by 2025 and achieving the Sustainable Development Goals by 2030 (2, 4). Thus, addressing child malnutrition necessitates designing multidisciplinary and multisectoral approaches to steer the development of national policies aimed at refocusing countries on the elimination path.

In sub-Saharan Africa, the prevalence of stunting at the sub-region and country levels has remained persistently high with a threshold >30% for most of the countries (2, 5, 6). In Rwanda, emerging evidence suggests that one in every three children is stunted (7), with sub-regional disparities in its distribution also reported (8). Reducing the prevalence of under five stunting remains a priority in Rwanda. To achieve this, several programs and policies have been developed and implemented through a public-private partnership. Notable programs include the USAID/*Gikuriro Kuri Bose* (Inclusive Nutrition and Early Childhood Development) and *Isoko y'Ubuzima* (The Thrive WASH) and the health systems strengthening policies including the community-based health insurance plan (*Mutuelles de Santé*), incorporation of community health workers into the healthcare system and performance-based financing of health care facilities (9, 10). However, stunting persists, increasing the need to understand the factors perpetuating stunting to recast policy decision-making and design more specific actions to address it.

Given the limited availability of resources needed to address various health challenges in Rwanda, evidence of key drivers of stunting among under-five children remain critical in designing effective and sustainable programs for addressing stunting. Earlier studies determining the prevalence of stunting in Rwanda have used DHS data. Due to the high geographical level analysis used in these studies, there may have been potential masking of local level variations that may be vital in understanding the effect of both geographical locations, and socio-demographic and environmental factors influencing stunting. Using five model districts where the *Gikuriro Kuri Bose* project is being implemented, the current study examined individual (child's factors, maternal/household factors), and environmental factors associated with child stunting in Rwanda to provide nuance evidence for policymaking and program design to address child stunting in Rwanda.

## Methods

### Study design and setting

**Figure 1:**
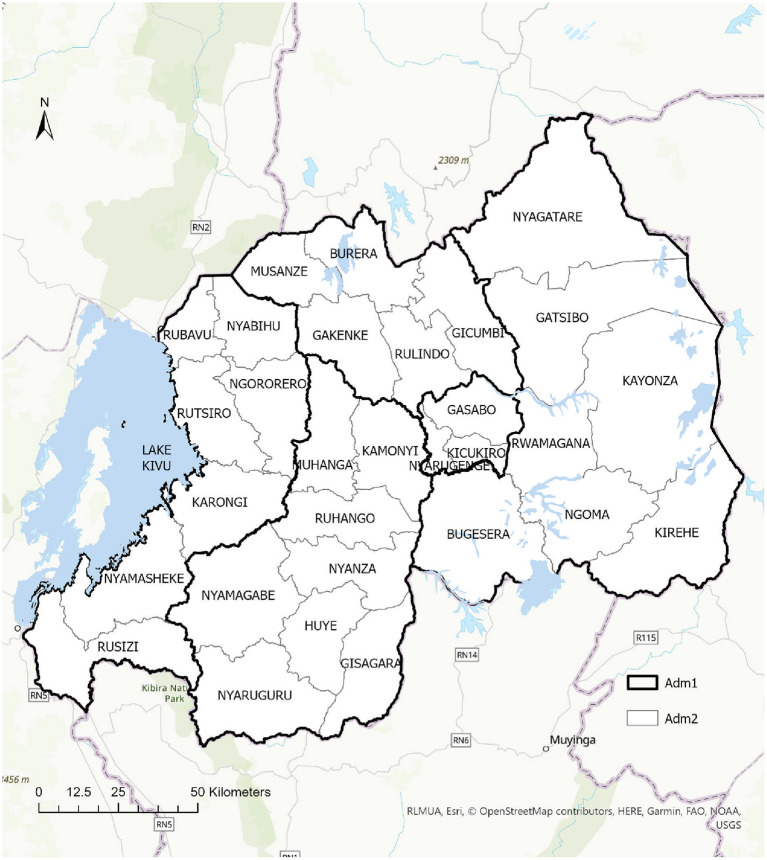
Map of Rwanda showing the administrative districts and project implementation areas.

This was a cross-sectional study conducted between September 6 and October 9 2022, in five districts of Rwanda including, Kicukiro, Ngoma, Burera, Nyabihu, and Nyanza. *Gikuriro Kuri Bose* is a multisectoral and transdisciplinary project being implemented in five districts of Rwanda, each being drawn from one province. Rwanda has four geopolitical provinces and the City of Kigali. The provinces and the City of Kigali are further subdivided into 30 districts and districts subdivided into sectors (416 sectors in total) and sectors subdivided into cells (2,148 cells) and cells subdivided into villages (14,837 villages). Villages comprise about 100 households while cells constitute between five-seven villages. The study districts included Nyabihu from the western province, Burera from the northern province, Kicukiro from the city of Kigali, Nyanza in the south and Ngoma in the eastern province (Figure 1).

### Data collection, sampling technique, and ethics

This study was based on 2,788 children and their mothers/legal guardians. To determine the sample size, the current prevalence of stunting (33%) (7) was considered as an indicator of the nutritional status. Using a 95% confidence interval and the equation proposed by Lwanga et al. (11) as *n* = $Z_{1-\frac{a}{2}}^{2}$ *(1-p)/* ε^2^*p*, where *p* = prevalence, ε = relative precision, and *n* = sample size with a relative precision for the study to be between 5 and 10% of the true prevalence (0.05 < ε < 0.10), a sample size of 713–2,854 pairs of mothers/guardians and children as adequate. From each household, children under five and their legal guardians were selected for inclusion in the study. In this study, the sampling unit was a cell. To obtain a representative sample, the study used a two-stage probabilistic sampling method. The first stage involved the random selection of cells from the sector and the second stage involved a systematic sampling of households from the selected cells.

Approval to conduct the study was granted by the University of Global Health Equity Institutional Review Board (UGHE-IRB: Ref: UGHE-IRB/2022/034). Furthermore, legal guardians of children were asked for consent, and this was provided in writing.

### Study variable

#### Dependent and independent variables

The dependent variable in this study was stunting, and this was a categorical binary variable (yes = 1 or no = 0). Stunting was defined as height for age *z*-score <-2 standard deviations using the WHO growth standards (12). Furthermore, using WHO classifications, children with height for age z-score of ≤-2 standard deviations and ≥-3 standard deviation were classified as moderately stunted while those with height for age *z*-score <-3 standard deviations were classified as severely stunted (13). There were three levels of the independent variables. These were categorized as individual (child and maternal/household) characteristics, community and environmental factors which included topography of the area, water, hygiene, and sanitation variables. To collect this information, a structured pre-tested questionnaire was administered to mothers/legal guardians of the children who had been included in the study. The questionnaire collected information on the child's age, sex, maternal/guardian's age, level of education, socio-economic class also called *Ubudehe*, breastfeeding and complementary feeding practices, hygiene and handwashing practices, household water availability and access, availability, and types of sanitary facilities, and socio-economic characteristics of the household. Additionally, information about the guardian and child's illnesses and disabilities (yes = 1 or no = 0) was collected. The classification of *Ubudehe* in Rwanda has been explained further in Supplementary material 1.

The weight of the children was measured using the SECA electronic scales to a precision of 0.1 kg while the height was taken to the nearest 0.1 cm using a UNICEF height/length board. To measure the height, children between the age of 24–59 months were made stand-upright without shoes and their height was taken using a stadiometer in a Frankfurt vertical position and to the nearest 0.1 cm. For children aged 0–23 months, their height/length was taken using a vertical measuring board while in a horizontal position. Before the measurements, it was ensured that the head, shoulders, and buttocks touched the board. To ascertain the validity of the anthropometry measurements, duplicate measurements were done for 10–15% of the sample and the variations for the duplicate measurements were below 5%. The age of the children was obtained from the *Ifishi Y'Ubuzima Bw'umwana* (vaccination card). The study included children aged between 0 and 59 months who were attending routine hospital outpatient visitations. Furthermore, the study included those without medical complications and those whose legal guardians consented to participate and signed the consent forms. All children in this age category but not fulfilling the inclusion criteria were excluded from the study.

To enhance the precision of the measurements, the SECA weighing scales were calibrated daily before the commencement of data collection. All data collectors were trained in the taking of child anthropometric measurements and administration of the face-to-face questionnaire interviews before data collection. Community health workers who were part of the data collection teams assisted with the taking of anthropometric measurements on all children. For children who could not be weighed on the SECA scale, the weight of the mother/legal guardian was initially taken. Thereafter, the weight of the mother/legal guardian while holding the child was taken. The difference between the two weights was taken as the weight of the child.

### Data analysis

Descriptive analysis was used to summarize continuous and categorical variables, showing their distribution with the outcome variable. The *Z*-score value for height-for-age was calculated using the ANTHRO PLUS software (14). In the bivariate and multivariate analysis, the response variable, stunting, was turned into a binary variable thus allowing us to logistic models. To determine the relationship between the various individual, community and environmental factors, a bivariate analysis was used. A multivariate multilevel logistic regression was used to examine the individual, community and environmental factors associated with under-five stunting. The multilevel models were deemed suitable for the analysis because of the hierarchical structure of the data and its ability to allow for the determination of the residual components associated with each level of the hierarchy. Furthermore, the multilevel models also allow for the estimation of group-level variables while estimating the group effects.

Three models were fit in the overall analysis. The first model was a null model, and this included the response variable only without any predictor variable and this was done to estimate its variance. In the second model which was a fixed effects model, we controlled for individual-level variables, and this included the children's demographic characteristics, history of diarrhea, breastfeeding and complementary feeding practices and child morbidity. In this model, district and sector were added as random intercept terms. Maternal (legal guardian) variables included education level and feeding structure, age and morbidity and water, hygiene, and environmental variables such as sanitation practices were also included. District and place of residence were added as random effects. The final model included both individual and contextual level factors which were the place of residence and district. The results demonstrating measures of association have been presented as adjusted odds ratios (aOR) together with their corresponding 95% confidence intervals (CIs) and *p*-values. The intraclass correlation coefficient (ICC), median odds ratio (MOR) and proportional change in variance (PCV) were used as a measure of the random effect. The ICC, which shows the proportion of total variance in the outcome attributable to districts, sectors and cells was calculated as shown by Merlo et al. (15). MOR is the measure of heterogeneity, and the PVC is the measure of the total variation of stunting in the final model (models with individual and environmental variables) comparative to the null model and was determined as described elsewhere (16, 17). Data analysis was carried out using StataSE STATA version 17 (StataCorp, College Station, TX, USA).

## Results

### Individual (child/maternal/household) level characteristics of study participants

Table 1 shows the child and maternal/household characteristics. The mean age (±SD) of the children was 26.4 (±16.2) months. The majority (*n* = 1,541, 55.3%) were aged between 24 and 59 months while only 7.6% (*n* = 211) were aged 0–5 months. In terms of gender, 50.4% (*n* = 1,404) of children were female while 49.6% (*n* = 1,384) were males. About 23.6% (*n* = 659) of the children had suffered from diarrhea 1–2 weeks before the study and of these, diarrhea lasted for 1–7 days among 93.6% (*n* = 617). Child characteristics; Child gender (*p* = 0.003), children with disability (*p* = 0.018), child age (*p* < 0.001) and morbidity factors; suffering from diarrhea (*p* < 0.001) were observed to be associated with stunting (Table 1).

**Table 1:** Prevalence of childhood stunting at various individual (child/maternal and household) level characteristics.

**Variable**	**Normal (*n*, %)**	**Stunted (*n*, %)**	***p-*** **value**
**Gender of child**			
Male	914 (66%)	470 (34%)	0.003
Female	1,001 (71.3%)	403 (28.7%)	
**Child disability**			
No	1,898 (68.9%)	856 (31.1%)	0.018
Yes	17 (50%)	17 (50%)	
**Child age category**			
0–11 months	467 (83%)	26 (17%)	0.001
12–23 months	443 (64.7%)	242 (35.3%)	
24–47 months	756 (65.5%)	398 (34.5%)	
48–59 months	249 (64%)	138 (36%)	
**Number of people in a family**			
1–5	1,380 (71%)	574 (29%)	0.001
> 6	535 (64.2%)	299 (35.8%	
**Gender of guardian**			
Male	127 (63%)	75 (37%)	0.064
Female	1,788 (69%)	798 31%)	
**Age category of guardian**			
15–24 years	386 (71%)	157 (29%)	0.217
25–49 years	1,464 (68%)	678 (32%)	
50–78 years	65 (63%)	38 (37%)	
**Employment status of guardian**			
Farmers	1,151 (68%)	542 (32%)	0.38
Self employed	124 (73%)	46 (27%)	
Domestic worker	7 (63.6%)	4 (36.4%)	
Unemployed	402 (71%)	164 (29%)	
Others	231 (66%)	117 (34%)	
**Socio-economic class (*****Ubudehe*** **category)**			
Category 1	183 (66%)	93 (34%)	0.157
Category 2	1,092 (68%)	515 (32%)	
Category 3	584 (70%)	250 (30%)	
No category/don't know	53 (79%)	14 (21%)	
**Guardian marital status**			
Never married	190 (69.6%)	83 (30.4%)	0.054
Married	758 (71.5%)	302 (28.5%)	
Formerly married	136 (65%)	73 (35%)	
Co-habiting	831 (66.7%)	415 (33.3%)	
**Guardian education level**			
None	376 (66%)	193 (34%)	0.001
Primary	1,073 (66.8%)	534 (33.2%)	
Secondary	437 (75.6%)	141 (24.4%)	
Tertiary	29 (85.3%)	5 (14.7%)	
**Guardian living with disability**			
No	1,887 (69%)	853 (31%)	0.119
Yes	28 (58%)	20 (42%)	
**Exclusive breastfeeding**			
No	948 (65%)	504 (35%)	0.001
Yes	967 (72%)	369 (28%)	
**Child with diarrhea in the past 2 weeks**			
No	1,500 (70.5%)	629 (29.5%)	0.001
Yes	415 (63%)	244 (37%)	
**Guardian access breastfeeding information**			
No	620 (66.5%)	312 (33.5%)	0.081
Yes	1,295 (69.8%)	561 (30.2%)	
**Child given vitamin A**			
No	538 (74.9%)	180 (25.1%)	0.001
Yes	1,377 (66.5%)	693 (33.5%)	
**Child given multinutient powder**			
No	1,574 (70%)	685 (30%)	0.02
Yes	341 (64.5%)	188 (35.5%)	
**Who feeds the child (complementary feeding)**			
Mother	1,657 (69.3%)	733 (30.7%)	0.154
Siblings	131 (63%)	77 (37%)	
Caretaker	105 (67%)	51 (33%)	
**Schedule of child complementary feeding**			
Childs demand	681 (73%)	258 (27%)	0.001
According to schedule	489 (71%)	199 (29%)	
Caretaker availability	55(73%)	20 (27%)	
Availability of food	690 (64%)	396 (36%)	
**Child feeding from own or communal plates**			
Communal plate	607 (70%)	261 (30%)	0.341
Own plate	1,308 (68%)	612 (32%)	
**Maternal morbidity**			
No	1421 (70%)	620 (30%)	0.078
Yes	494 (66%)	253 (34%)	

The median age (±IQR) of the guardians/mothers was 30 (±11) years. Most of the guardians (*n* = 1,607, 57.6%) had primary education. In terms of marital status, 44.7% (*n* = 1,246) were cohabiting while 38% (*n* = 1,060) were married. The median number of people in the households was 5 (±2) and most households (*n* = 2,258, 81%) had one child below the age of five. Furthermore, 1.7% (*n* = 48) of the guardians were living the disabilities and in terms of the *Ubudehe* category, 57.7% (*n* = 1,607) were in category 2. In addition, 47.9% (*n* = 1,336) exclusively breastfed their children and of these, 78.9% (*n* = 1,054) breastfed on the child's demand while 5.1% (*n* = 68) breastfed according to a schedule. Maternal and household characteristics such as the number of people living in a household (*p* = 0.001), number of under-five children in a household (*p* = 0.004), maternal level of education (*p* < 0.001), exclusive breastfeeding (*p* < 0.001), complementary feeding (*p* < 0.001), and frequency of complementary feeding (*p* < 0.001) were observed to be associated with stunting (Table 1).

About 52.4% (*n* = 1,461) had tap-pipped water as their main drinking water source while 2.2% (*n* = 65) relied on water tankers. Furthermore, most households (*n* = 1,690) did not treat their water. In terms of monthly water availability, 68.2% (*n* = 1,899) did not have adequate water while 31.8% (*n* = 889) had adequate water. In terms of sanitation, 57.8% (*n* = 1,610) used pit latrines with concrete floors while 2.9% (*n* = 80) used open defecation. In addition, 25.2% (*n* = 701) were sharing sanitation facilities with non-family members. For maternal handwashing practices, 50.1% (*n* = 1,397) reported to have been washing their hands before eating and 39.6% (*n* = 1,103) before eating and feeding the child. Source of main drinking water (*p* < 0.001), time taken to fetch water (*p* < 0.001), water treatment (*p* = 0.027) and type of facility used (*p* = 0.005) were associated with stunting (Table 2).

**Table 2:** Prevalence of childhood stunting at various environmental and WASH characteristics.

**Variable**	**Normal (*n*, %)**	**Stunted (*n*, %)**	***p-*** **value**
**Parents hand washing practices**			
Before eating	958 (69%)	439 (31%)	0.964^**^
Before eating and feeding the baby	763 (69%)	340 (31%)	
Before eating, feeding the baby and toilet use	12 (67%)	6 (33%)	
Before eating and after toilet use	21 (70%)	9 (30%)	
Before feeding the baby	75 (65%)	41 (35%)	
Before feeding the baby and after toilet use	3 (60%)	2 (40%)	
After toilet use	83 (70%)	36 (30%)	
**Toilet sharing**			
No	1,443 (69%)	644 (31%)	0.347
Yes	472 (67%)	229 (33%)	
**Toilet facility available**			
Pour flash	45 (80%)	11 (20%)	0.005
Pit latrine with slab	1,121 (70%)	489 (30%)	
Pit latrine no slab	706 (68%)	336 (32%)	
Open defecation	43 (54%)	37 (46%)	
**Water availability**			
No	1,322 (69%)	583 (31%)	0.191
Yes	595 (67%)	294 (33%)	
**Time spent fetching**			
0–30 min	1,499 (70%)	639 (30%)	0.010
31–60 min	338 (64%)	193 (36%)	
61–180 min	74 (65%)	40 (35%)	
**Source of drinking water**			
Tap water	1,051 (72%)	410(28%)	0.001
Borehole	583 (63%)	344 (37%)	
Water tanker	43(66%)	22(34%)	
River/lake/rainwater	238(71%)	97(29%)	
**Topography/terrain of the area**			
Highland	305 (64%)	171 (36%)	0.059
Flat terrain	1,162 (70%)	505 (30%)	
Low land	448 (69%)	197 (31%)	

** Fishers exact tested used.

**Figure 2:**
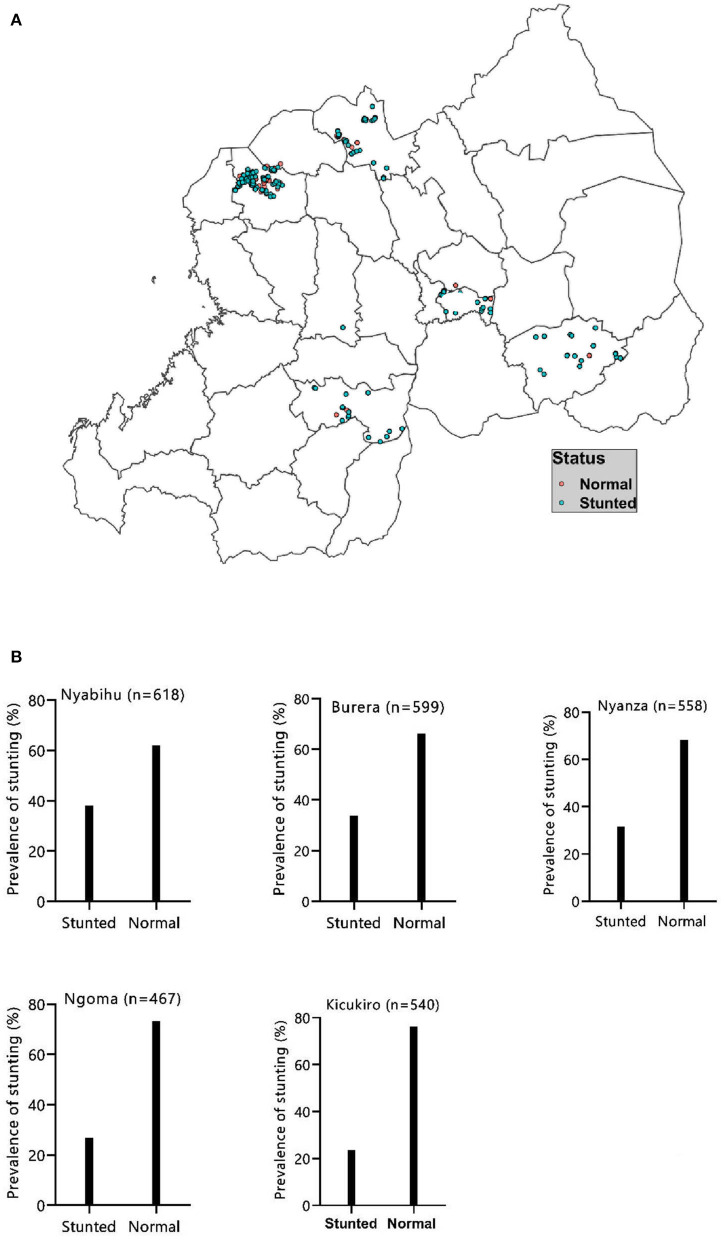
Distribution **(A)** and proportion **(B)** of childhood stunting in the study areas.

Figure 2A shows the prevalence of stunting in the five study sites. The prevalence of stunting was 31.4% (95% CI: 29.5–33.1). Of this, 12.2% were severely stunted while 19.2% were moderately stunted. Stunting was high in Nyabihu (37.8%) followed by Burera (34.1%) and was least in Kicukiro (23.8%) (Figure 2B).

### Multilevel modeling

The results from the multilevel models show that individual and environmental level factors influenced stunting (Table 3). In the final multilevel model controlling for factors, individual-level factors, female children were about 76% (aOR: 0.76, 95% CI: 0.64–0.90) less likely to be stunted compared to male children. Furthermore, the odds of stunting increased with age; children aged 24–47 months were 3.6 (95% CI: 2.00–6.49) more likely to be stunted compared to those <6 months of age. Also, children who came from households where they were at least two children below the age of five (aOR: 1.29; 95% CI: 1.02–1.62) and those coming from families with more than six people (aOR: 1.35; 95% CI: 1.10–1.66) were more likely to be stunted than those from households with 1–5 people. In addition, children who reported having had diarrhea 2 weeks before the study (aOR: 1.32; 95% CI: 1.07–1.62) and those who fed themselves from their plates (aOR: 1.45; 95% CI: 1.19–1.78) were also more likely to be stunted (Table 3). Among the environmental factors, the use of tap water reduced the odds of stunting by 75% (95% CI: 0.59–0.94) while children from households that had no sanitation facilities (aOR: 3.35; 95% CI: 1.38–8.10) were more likely to be stunted than those who had flush pour toilets (Table 3). Among the contextual factors, children from Nyabihu (aOR: 1.95; 95% CI: 1.33–2.86) and Burera (aOR: 1.77; 95% CI: 1.16–2.71) were more likely to be stunted than those from Kicukiro.

**Table 3:** Factors associated with childhood stunting in a multilevel logistic regression.

**Variables**	**Model 0**	**Model I** **aOR (95%CI)**	**Model II** **aOR (95%CI**	**Model III** **aOR (95%CI)**
**INDIVIDUAL FACTORS**				
**Gender of child**				
Male		**1**		**1**
Female		0.76^**^ (0.64–0.90)		0.76^**^ (0.64–0.90)
**Age in months**				
0–11		**1**		1
12–23		2.65^***^ (1.57–4.46)		2.77^***^ (1.64–4.67)
24–47		3.42^***^ (1.90–6.17)		3.60^***^ (2.00–6.49)
48–59		3.31^***^ (1.79–6.14)		3.51^***^ (1.89–6.50)
**Child has disability**				
No		**1**		**1**
Yes		2.07^*^ (1.00–4.26)		2.06^*^ (1.00–4.25)
**Number of people in household**				
1–5		**1**		1
>6		1.33^**^ (1.09–1.64)		1.35^**^ (1.10–1.66)
**Number of children under 5 years**				
One		**1**		1
Two		1.30^*^ (1.03–1.64)		1.29^*^ (1.02–1.62)
Three		1.81 (0.83–3.95)		1.89 (0.86–4.14)
**Gender of legal guardian**				
Male		**1**		**1**
Female		0.96 (0.68–1.37)		0.99 (0.70–1.41)
**Age of legal guardian**				
15–24		**1**		**1**
25–49		1.12 (0.88–1.44)		1.13 (0.88–1.45)
50–78		1.01 (0.58–1.78)		1.03 (0.58–1.80)
**Employment status of guardian**				
Farmers		**1**		**1**
Self employed		0.87 (0.58–1.29)		0.89 (0.59–1.33)
Domestic worker		1.37 (0.37–5.14)		1.41 (0.38–5.27)
Unemployed		0.92 (0.71–1.20)		0.94 (0.71–1.24)
Others		1.12 (0.84–1.49)		1.14 (0.85–1.53)
**Socio-economic category**				
Category 1		**1**		1
Category 2		1.07 (0.79–1.46)		1.07 (0.79–1.45)
Category 3		0.97 (0.79–1.35)		0.98 (0.71–1.36)
No category given/don't know		0.70 (0.79–1.40)		0.71 (0.35–1.42)
**Guardian marital status**				
Never married		**1**		1
Married		0.83 (0.59–1.17)		0.82 (0.58–1.16)
Cohabiting		1.16 (0.79–1.61)		1.13 (0.81–1.57)
Formerly married		1.05 (0.79–1.65)		1.05 (0.67–1.65)
**Guardian level of education**				
None		**1**		1
Primary		1.17 (0.93–1.48)		1.17 (0.93–1.47)
Secondary		0.83 (0.61–1.12)		0.81 (0.60–1.09)
Tertiary		0.50 (0.18–1.46)		0.54 (0.19–1.52)
**Guardian has disability**				
No		**1**		**1**
Yes		1.27 (0.67–2.42)		1.24 (0.65–2.35)
**Breastfeeding child**				
No		**1**		1
Yes		0.86 (0.63–1.19)		0.87 (0.63–1.20)
**Child had diarrhea in past 2 weeks**				
No		**1**		**1**
Yes		1.32^**^ (1.07–1.62)		1.32^**^ (1.07–1.62)
**Mother has access to breastfeeding information**				
No		**1**		**1**
Yes		0.96 (0.79–1.16)		0.96 (0.79–1.16)
**Child given Vitamin A at 6 months**				
No		**1**		**1**
Yes		1.11 (0.87–1.42)		1.10 (0.86–1.41)
**Use of multinutient powder**				
No		**1**		**1**
Yes		1.16 (0.92–1.46)		1.13 (0.90–1.42)
**Who feeds baby**				
Siblings		**1**		**1**
Mother		0.91 (0.66–1.27)		0.93 (0.67–1.29)
Caretaker		0.98 (0.59–1.61)		1.00 (0.61–1.65)
**When is baby given food**				
Child's demand		**1**		**1**
Schedule		0.95 (0.75–1.22)		0.94 (0.74–1.20)
Caretaker availability		0.72 (0.41–1.28)		0.67 (0.38–1.19)
Availability of food		1.06 (0.85–1.34)		1.04 (0.82–1.30)
**Eating plate used by**				
Common plate		**1**		**1**
Own plate		1.45^***^ (1.18–1.78)		1.45^***^ (1.19–1.78)
**Maternal illness recorded**				
No		**1**		**1**
Yes		1.09 (0.89–1.33)		1.09 (0.89–1.32)
**Parents washing their hands**				
Before eating		**1**		**1**
Before eating and before feeding child		0.99 (0.81–1.21)		0.97 (0.79–1.19)
Before feeding		1.41 (0.90–2.21)		1.43 (0.91–2.25)
Before feeding child and after toilet		1.29 (0.86–1.93)		1.31 (0.87–1.97)
**Toilet sharing**				
No		**1**		**1**
Yes		1.11^***^ (0.89–1.37)		1.05^**^ (1.12–1.43)
**Type of toilet used**				
Pour flash		**1**		**1**
Latrines with slab		2.02 (0.96–4.22)		2.06 (0.98–4.32)
Latrines with no slab		1.99 (0.94–4.23)		2.05 (0.96–4.38)
Open defecation		3.40^**^ (1.41–4.21)		3.35^**^ (1.38–8.10)
**Family had adequate water last month**				
No		**1**		**1**
Yes		1.06 (0.87–1.29)		1.04 (0.85–1.27)
**Water treatment method**				
Nothing		**1**		**1**
Boiling		0.66^**^ (0.48–0.90)		0.66^*^ (0.49–0.91)
Add chemicals		0.84 (0.69–1.02)		0.83 (0.68–1.01)
Filter		1.99 (0.61–6.50)		2.20 (0.67–7.23)
Others		0.93 (2.44–3.57)		1.01 (0.26–3.87)
**Time spent fetching water**				
0–30 min		**1**		**1**
31–60 min		1.22 (0.97–1.53)		1.20 (0.95–1.52)
61–180 min		1.08 (0.69–1.69)		1.04 (0.66–1.62)
**Main source of family drinking water**				
Borehole		**1**		**1**
Water tank		0.91 (0.50–1.66)		0.93 (0.51–1.70)
Tap water		0.73^**^ (0.58–0.94)		0.75^*^ (0.59–0.94)
River/lake/rainwater		0.91 (0.41–2.01)		0.92 (0.41–2.02)
**CONTEXTUAL LEVEL FACTORS**				
**Place of residence**				
Flat terrain			**1**	**1**
Highland			0.99 (0.74–1.31)	0.90 (0.66–1.22)
low land			0.91 (0.71–1.15)	1.00 (0.77–1.29)
**District**				
Kicukiro			**1**	**1**
Burera			1.65^**^ (1.13–2.40)	1.77^**^ (1.16–2.71)
Ngoma			1.15 (0.78–1.68)	1.19 (0.78–1.81)
Nyabihu			2.01^***^ (1.40–2.88)	1.95^***^ (1.33–2.86)
Nyanza			1.43 (0.98–2.07)	1.21 (0.79–1.84)
**Random effects**				
Variance (CI)	0.10 (0.04–0.28)	0.09 (0.03–0.28)	0.09 (0.03–0.27)	0.08 (0.03–0.28)
ICC (%)	4.4	3.7	4.0	3.6
PCV (%)	Ref	10.0	10.0	20.0
MOR	1.35	1.32	1.34	1.32
**Model fitness**				
Log-likelihood	−1,715.1	−1,707.7	−1,714.1	−1,598.8
AIC	3,436.1	3,311.9	3,440.2	3,310.6
N	2,788	2,788	2,788	2,788

*** *p* < 0.001;

** *p* < 0.01;

* *p* < 0.05;

1 = reference category; Model 0 contains no explanatory variables; Model I included individual-level factors only; Model II included community-level factors only; Model III included both individual-level and community-level factors: aOR, adjusted odds ratio; CI, confidence internal; ICC, intraclass correlation coefficient; PVC, proportional variance change; AIC, akaike information criterion; BIC, Bayesian information criterion.

Measures of variations. The bold values are just reference values for the models.

On the measures of variation in the random effect, the results of the Akaike Information Criterion (AIC), Intraclass Correlation Coefficient (ICC), Median Odds Ratio (MOR), and proportional change in variance (PCV) suggest that the final model best fit the data. In the null model, ICC indicated that 4.4% of the variance in stunting was attributed to the community or contextual factors and this was reduced to 3.6% in the final model. In the final model, as shown by the PVC, 20% of the variance in the odds of stunting was accounted for by the model (Table 3).

## Discussion

This study sought to understand the individual and environmental determinants of stunting in Rwanda using data collected from a cross-sectional study of five districts. The study has shown that both individual and community-level factors are critical in determining the linear growth of children. The fit model further showed that 20% of the variation in stunting was accounted for by individual-level factors, environmental-level factors, and contextual-level factors such as place of residence. Stunting increased with age, with children aged 24–59 months having the highest odds of stunting. This finding corroborates pooled results from East Africa (18), Rwanda (1, 19), Ethiopia (20), and Kenya (21) and elsewhere (22) which observed an increase in the risk of stunting with age before a subsequently reduced after the age of 48 months. The observed rise in the risk of stunting with age in our study as well as others may be due to reduced milk intake and weaning of children together with increased risks of infection because of increased exposure of children to unhygienic environments (18, 23).

Several earlier studies (1, 8, 24, 25) including ours have reported child gender as being an important determinant of stunting. Our results as well as those by Mzumara et al. (25), Binagwaho et al. (1), and Adekanmbi et al. (24) have shown that male children are more likely to be stunted as compared to female children. According to several authors, growth among male children is affected by repeated respiratory infections due to slower lung maturation (26), cultural norms due to the usefulness of women in agricultural activities (27) and potentially hormonal and genetic determinants (20). Elsewhere, the gender-based differences in stunting have been suggested even after controlling for gestational age and body size, increased risks of morbidity among male children predispose them to stunt (28) and a higher proportion of male preterm births as compared to female preterm births (29) thus increasing the likelihood of stunting among male children. The current study further observed that stunting was higher among households where the family size had more than six people, those with at least two children under the age of five and children who were fed from communal or family food portions. This may be due to the reduced quality of life for children (25); the likelihood of potential struggle for nutrition (30) due to families' inability to meet the dietary requirements and inadequate healthcare-related services for children and other family members.

Our study shows that children who reported diarrhea 2 weeks before the study, those from households with no toilets and those that shared toilets had higher odds of stunting. Furthermore, of the environmental factors studied, the results suggested that the lack of sanitary facilities increased the risks of stunting, an observation corroborated by others (31, 32). This association may be linked to open defecation resulting in fecal contamination of food and water, especially untreated water which fuels diarrheal illness and reduces the rate of attainment of developmental milestones among children. Furthermore, Crocker and Bartram (31) and Modern et al. (32) suggested that sharing toilets among different households increased the risk of diarrhea in children thus increasing their odds of stunting. Other studies focusing on sanitation and stunting among children concluded that poor water, hygiene, and sanitation facilities may lead to diarrhea and intestinal worm infections (33, 34) affecting the nutritional status of children. The finding from our study and those of other authors indicate the importance of sanitation in improving child growth. Furthermore, our results have shown a high prevalence of some health behaviors such as handwashing and sanitation-related such as use of toilets. Thus, there is need to build on these behaviors by focusing on increasing access to piped and treated water. Also, designing of sustainable water and sanitation-related activities and including various community engagement activities would be vital in strengthening the community's ability to manage water and sanitation facilities. Furthermore, enhancing community health education and exposure to health messages promoting hygiene practices would be critical in reducing the risks of diarrhea among children.

This study used the multilevel method of analysis to understand the effect of socio-demographics, environmental determinants, and community-level factors on stunting in Rwanda. By using this approach, it was made possible to understand how community-level factors such as place of residence influence stunting. The results from the study show that community-level factors can interact with individual-level factors to influence stunting among children. Earlier studies by Adekanmbi et al. (24) and Frohlich et al. (35) applied similar methods in understanding and differentiating the contributions of community and individual level factors on the variation in the outcome variable. The results of the current study demonstrate that both individual-level and community-level factors are associated with childhood stunting. Furthermore, community-level factors account for more variation in stunting above than individual-level factors. This, suggests that community-level factors have a significant influence on stunting and this observation was also made by Pickett and Pearl (36).

Reducing stunting and addressing other undernutrition indicators necessitates understanding and altering several underlying factors. Although several programs and policies have been implemented, there is a need to address nutritional security in a broader context. For instance, the provision of food interventions without addressing individual and environmental factors such as maternal education and socio-economic and health wellbeing, household sanitation and clean water, and access to adequate and quality health services would only lead to short or medium terms achievements in stunting reduction. An earlier study suggested that various factors that influence stunting interact in a complex and diverse way (37). Thus, cross-disciplinary and multisectoral approaches are vital in addressing it. For instance, programs or interventions meant to address factors influencing stunting would need to involve expertise from nutritionists to deal with child and maternal nutrition and feeding practices, healthcare services to manage poor health conditions, environmentalists to help prevent and control environmental contamination that may cause illnesses, while health promotion would be added to provide continuous health education to the community members. The results from the current study suggest that addressing stunting in Rwanda requires addressing both individual and environmental factors. While the government provides the framework for addressing these factors, it's important to design programs that incorporate strategies that address the problem in the short and medium terms and build on the short and medium-term achievements to devise long-term strategies. In addition, designing programs that target groups at high risk such as children older than 11 months and children from larger families can maximize outcomes.

## Strengths and limitations

Unlike previous studies done in Rwanda, this study used a multilevel analysis which made it possible to identify factors influencing stunting at both individual and environmental levels. Furthermore, the high response rate from the participants drawn from different districts in the five provinces ensured that most information was captured. On the other hand, our study had the limitation of being cross-sectional thus it may be difficult to ascertain the causal relationship.

## Conclusion

Addressing and preventing childhood stunting is crucial in averting potential future health, cognitive and economic development of the country and nations at large. The finding from the current study shows that stunting is prevalent and remains a public health challenge in Rwanda. This study suggests the need to design cross-sectoral and transdisciplinary approaches to address stunting. Furthermore, sustainable approaches addressing child's health and nutrition in long term should target places with a high rate of stunting, high-risk groups such as children from large households and enhance maternal health education. The current study further suggests the need to conduct in-depth qualitative studies to explore and identify reasons for the high levels of stunting in parts of Rwanda, especially the Northern and North-western where the prevalence of stunting is higher than in other regions.

## Data availability statement

The raw data supporting the conclusions of this article will be made available by the authors, without undue reservation.

## Ethics statement

The studies involving human participants were reviewed and approved by University of Global Health Equity Institutional Review Board (Ref: UGHE-IRB/2022/034). Written informed consent to participate in this study was provided by the participants' legal guardian/next of kin.

## Author contributions

CK, RW, AG, and AB conceptualized the study. CK and MP conducted the statistical analysis. CK wrote the manuscript. MQ and MI were involved in conceptualizing the study and carried out data extraction. RW, AB, and AG sourced for funding and contributed toward editing the manuscript. All authors have read and approved the final manuscript.

## Supplementary material

Supplementary material 1 Classification of the socio-economic (*Ubudehe*) in Rwanda.

## References

[1] Binagwaho A Rukundo A Powers S Donahoe KB Agbonyitor M Ngabo F. Trends in burden and risk factors associated with childhood stunting in Rwanda from 2000 to 2015: policy and program implications. BMC Public Health. (2020) 20:83. 10.1186/s12889-020-8164-4 31959142 PMC6971879

[2] UNICEF, WHO, World Bank Group. Levels and Trends in Child Malnutrition: Towards a Future Without Malnutrition for Every Child. Report No.: 9240025251. UNICEF/WHO/World Bank Group Joint Child Malnutrition Estimates (2021).

[3] Assembly UG. Universal declaration of human rights. UN General Assembly. (1948) 302:14–25.

[4] Amoroso L. Post-2015 agenda and sustainable development goals: where are we now? Global opportunities to address malnutrition in all its forms, including hidden hunger Hidden Hunger. Strat Improve Nutr Q. (2018) 118:45–56. 10.1159/000484334 33503779

[5] Quamme SH Iversen PO. Prevalence of child stunting in Sub-Saharan Africa and its risk factors. Clin Nutr Open Sci. (2022) 42:49–61. 10.1016/j.nutos.2022.01.009

[6] Ssentongo P Ssentongo AE Ba DM Ericson JE Na M Gao X. Global, regional and national epidemiology and prevalence of child stunting, wasting and underweight in low- and middle-income countries, 2006-2018. Sci Rep. (2021) 11:5204. 10.1038/s41598-021-84302-w 33664313 PMC7933191

[7] NISR, Ministry of Finance Economic Planning/Rwanda, Ministry of Health/Rwanda ICF International. Rwanda Demographic and Health Survey 2019-20. Kigali; Rockville, MD: NISR/MOH/ICF (2021).

[8] Uwiringiyimana V Veldkamp A Amer S. Stunting spatial pattern in Rwanda: an examination of the demographic, socio-economic and environmental determinants. Geospat Health. (2019) 14:329–39. 10.4081/gh.2019.820 31724383

[9] Binagwaho A Scott KW. Improving the world's health through the post-2015 development agenda: perspectives from Rwanda. Int J Health Policy Manag. (2015) 4:203. 10.15171/ijhpm.2015.46 25844381 PMC4380561

[10] Janssen W de Dieu Ngirabega J Matungwa M Van Bastelaere S. Improving quality through performance-based financing in district hospitals in Rwanda between 2006 and 2010: a 5-year experience. Trop Doct. (2015) 45:27–35. 10.1177/0049475514554481 25406257

[11] Lwanga SK Lemeshow S. Sample Size Determination in Health Studies: A Practical Manual. Geneva: World Health Organization (1991).

[12] WHO. WHO Child Growth Standards: Length/Height-for-Age, Weight-for-Age, Weight-for-Length, Weight-for-Height and Body Mass Index-for-Age: Methods and Development. Geneva: WHO (2006).

[13] WHO. Guideline: Assessing and Managing Children at Primary Health-Care Facilities to Prevent Overweight and Obesity in the Context of the Double Burden of Malnutrition: Updates for the Integrated Management of Childhood Illness (IMCI). Geneva: World Health Organization (2017). 29578661

[14] World Health Organization AnthroPlus. AnthroPlus: Software for Assessing Growth of the World's Children and Adolescents. Geneva: Department of Nutrition for Health and Development (2009).

[15] Merlo J Chaix B Ohlsson H Beckman A Johnell K Hjerpe P. A brief conceptual tutorial of multilevel analysis in social epidemiology: using measures of clustering in multilevel logistic regression to investigate contextual phenomena. J Epidemiol Commun Health. (2006) 60:290–7. English. 10.1136/jech.2004.029454 16537344 PMC2566165

[16] Halonen JI Kivimaki M Pentti J Kawachi I Virtanen M Martikainen P. Quantifying neighbourhood socioeconomic effects in clustering of behaviour-related risk factors: a multilevel analysis. PLoS ONE. (2012) 7:e32937. 10.1371/journal.pone.0032937 22427912 PMC3299718

[17] Larsen K Merlo J. Appropriate assessment of neighborhood effects on individual health: integrating random and fixed effects in multilevel logistic regression. Am J Epidemiol. (2005) 161:81–8. 10.1093/aje/kwi017 15615918

[18] Tesema GA Yeshaw Y Worku MG Tessema ZT Teshale AB. Pooled prevalence and associated factors of chronic undernutrition among under-five children in East Africa: a multilevel analysis. PLoS ONE. (2021) 16:e0248637. English. 10.1371/journal.pone.0248637 33765094 PMC7993805

[19] Nshimyiryo A Hedt-Gauthier B Mutaganzwa C Kirk CM Beck K Ndayisaba A. Risk factors for stunting among children under five years: a cross-sectional population-based study in Rwanda using the 2015 Demographic and Health Survey. BMC Public Health. (2019) 19:1–10. English. 10.1186/s12889-019-6504-z 30744614 PMC6371425

[20] Hailu BA Bogale GG Beyene J. Spatial heterogeneity and factors influencing stunting and severe stunting among under-5 children in Ethiopia: spatial and multilevel analysis. Sci Rep. (2020) 10:1–10. English. 10.1038/s41598-020-73572-5 33009463 PMC7532151

[21] Shinsugi C Matsumura M Karama M Tanaka J Changoma M Kaneko S. Factors associated with stunting among children according to the level of food insecurity in the household: a cross-sectional study in a rural community of Southeastern Kenya. BMC Public Health. (2015) 15:1–10. English. 10.1186/s12889-015-1802-6 25924925 PMC4428099

[22] Phiri M Mulemena D Kalinda C Odhiambo JN. Contextual factors and spatial trends of childhood malnutrition in Zambia. PLoS ONE. (2022) 17:e0277015. 10.1371/journal.pone.0277015 36327254 PMC9632925

[23] Daelmans B Ferguson E Lutter CK Singh N Pachon H Creed-Kanashiro H. Designing appropriate complementary feeding recommendations: tools for programmatic action. Matern Child Nutr. (2013) 9 Suppl 2:116–30. 10.1111/mcn.12083 24074322 PMC6860844

[24] Adekanmbi VT Kayode GA Uthman OA. Individual and contextual factors associated with childhood stunting in Nigeria: a multilevel analysis. Matern Child Nutr. (2013) 9:244–59. 10.1111/j.1740-8709.2011.00361.x 22004134 PMC6860873

[25] Mzumara B Bwembya P Halwiindi H Mugode R Banda J. Factors associated with stunting among children below five years of age in Zambia: evidence from the 2014 Zambia demographic and health survey. BMC Nutr. (2018) 4:1–8. English. 10.1186/s40795-018-0260-9 32153912 PMC7050779

[26] Vu HD Dickinson C Kandasamy Y. Sex difference in mortality for premature and low birth weight neonates: a systematic review. Am J Perinatol. (2018) 35:707–15. 10.1055/s-0037-1608876 29241280

[27] Sevedberg P. Undernutrition–gender bias in sub-Saharan Africa. J Devel Stud. (1996) 32:933–43. 10.1080/00220389608422447

[28] Elsmén E Pupp IH Hellström-Westas L. Preterm male infants need more initial respiratory and circulatory support than female infants. Acta Paediatr. (2004) 93:529–33. 10.1080/08035250410024998 15188982

[29] Escobar GJ Clark RH Greene JD. Short-term outcomes of infants born at 35 and 36 weeks gestation: we need to ask more questions. Semin Perinatol. (2006) 30:28–33. 10.1053/j.semperi.2006.01.005 16549211

[30] Servili C Medhin G Hanlon C Tomlinson M Worku B Baheretibeb Y. Maternal common mental disorders and infant development in Ethiopia: the P-MaMiE Birth Cohort. BMC Public Health. (2010) 10:693. 10.1186/1471-2458-10-693 21073710 PMC3091583

[31] Crocker J Bartram J. Interpreting the global enteric multicenter study (GEMS) findings on sanitation, hygiene, and diarrhea. PLoS Med. (2016) 13:e1002011. 10.1371/journal.pmed.1002011 27138924 PMC4854482

[32] Modern G Sauli E Mpolya E. Correlates of diarrhea and stunting among under-five children in Ruvuma, Tanzania; a hospital-based cross-sectional study. Sci Afr. (2020) 8:e00430. English. 10.1016/j.sciaf.2020.e00430

[33] Briend A. Is diarrhoea a major cause of malnutrition among the under-fives in developing countries? A review of available evidence. Eur J Clin Nutr. (1990) 44:611–28. 2261894

[34] Hall A Hewitt G Tuffrey V de Silva N. A review and meta-analysis of the impact of intestinal worms on child growth and nutrition. Matern Child Nutr. (2008) 4:118–236. English. 10.1111/j.1740-8709.2007.00127.x 18289159 PMC6860651

[35] Frohlich KL Potvin L Gauvin L Chabot P. Youth smoking initiation: disentangling context from composition. Health Place. (2002) 8:155–66. 10.1016/S1353-8292(02)00003-5 12135639

[36] Pickett KE Pearl M. Multilevel analyses of neighbourhood socioeconomic context and health outcomes: a critical review. J Epidemiol Community Health. (2001) 55:111–22. 10.1136/jech.55.2.111 11154250 PMC1731829

[37] Strauss J Thomas D. Health, nutrition, and economic development. J Econ Literat. (1998) 36:766–817. English.

