# Supplementary material for: Socio-demographic and environmental determinants of under-5 stunting in Rwanda: Evidence from a multisectoral study
Source: Front Public Health. 2023 Mar 14;11:1107300. doi: 10.3389/fpubh.2023.1107300 (PMC10043183; doi:10.3389/fpubh.2023.1107300)
Supplement: Supplementary material 1 — Classification of the socio-economic (Ubudehe) in Rwanda. [file Data_Sheet_1.docx]

Supplementary Material 1

**Socio-demographics and environmental determinants of under-5 stunting in Rwanda: Evidence from a multisectoral study**

*Corresponding Author: Chester Kalinda^1*^: [ckalinda@gmail.com](mailto:ckalinda@gmail.com), [ckalinda@ughe.org](mailto:ckalinda@ughe.org)

The Ubudehe categorization is a socioeconomic stratification system which was first established by the Government of Rwanda in 2000 as part of the strategies to address poverty. There were six Ubudehe poverty categories from inception until February 2015 when they were revised from six to four numerical naming being category 1, category 2, category 3 and category 4 (1), and then to five letters (A, B, C, D, E) from 2021 (2).

From 2015, each household of the Rwandan population was categorized into any one of the four Ubudehe categories with the first category (category 1) designated for the poorest people in society while the fourth category (category 4) was for the wealthiest members of society. About 4.6 million Rwandans or 46 per cent of the population were either in the first or second category (Government of Rwanda 2015, MINALOC 2015) (3).

With the current and new stratification using letters (A, B, C, D, E), Category A consists of households that are considered well-off such as families with an aggregated income of more than 600, 000 Rwf per month. The income sources can be from salaries or pension benefits, or other income-generating activities. Category B comprises households that earn between 65, 000 Rwf and 600, 000 Rwf monthly from similar sources as those mentioned in Category A. Furthermore, someone can be categorized in this class (Category B) if they are from the rural areas and own land measuring between one and 10 hectares or if they are from the urban areas and own between 300 square metres and one hectare. Category C consists of households that make an aggregated income of between 45, 000 Rwf and 65, 000 Rwf per month. Their land ownership ranges from 0.5 hectares to one hectare in rural areas, or 100 square metres to 300 square metres in urban areas. Category D is for households that earn less than 45, 000 Rwf a month (casual workers). Their land is less than half a hectare in rural areas, and less than 100 square metres in urban areas. Lastly, category E is a special category comprising people out of the labour force because of age, those with major disabilities or incurable diseases and do not own other assets or other sources of livelihood (2). Ubudehe categories are revised every three years and cover more than 2.7 million households (1).

**References**

1. Government L. Ministry of Local Government NATIONAL SOCIAL PROTECTION POLICY. 2020;

2. Bishumba N. Rwanda: New Ubudehe Categories to Be Activated By December. ? New Times [Internet]. 2021;1–10. Available from: https://www.newtimes.co.rw/news/new-ubudehe-categories-be-activated-december

3. Poverty level categories What are HGI. 2015;30.
